# Supplementary figures and images for: Comparative transcriptome analysis of differentially expressed genes related to the physiological changes of yellow-green leaf mutant of maize
Source: PeerJ. 2021 Feb 16;9:e10567. doi: 10.7717/peerj.10567 (PMC7894110; doi:10.7717/peerj.10567)

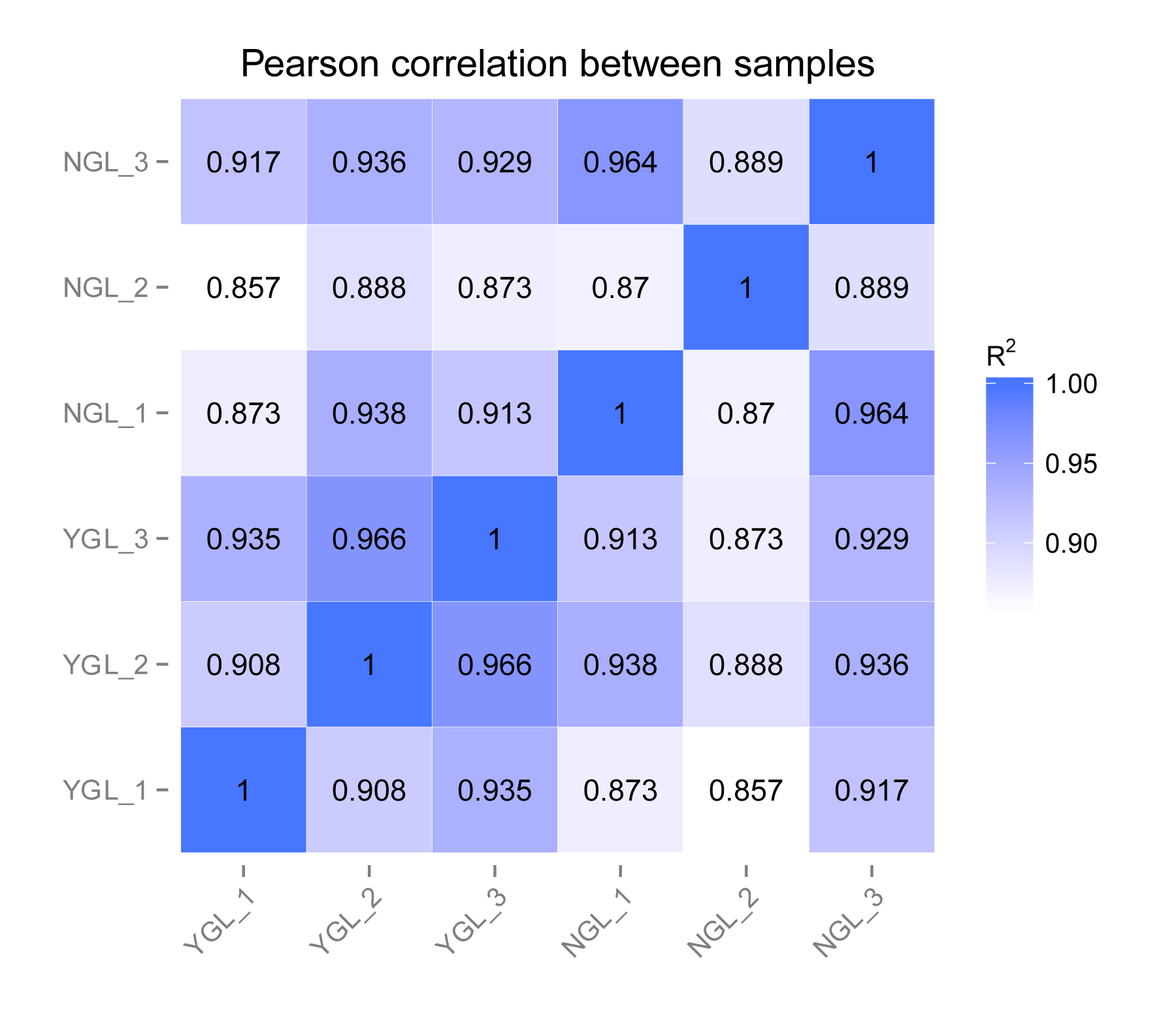

Supplement: Supplemental Information 7 — The R2 value of the Pearson correlation between each pair of samples is presented in the center of each square. NGL_1, NGL_2 and NGL_3 are the three repetitions of the normal green leaf inbred line. YGL_1, YGL_2 and YGL_3 are the three repetitions of yellow-green leaf mutant. [file peerj-09-10567-s007.jpg]

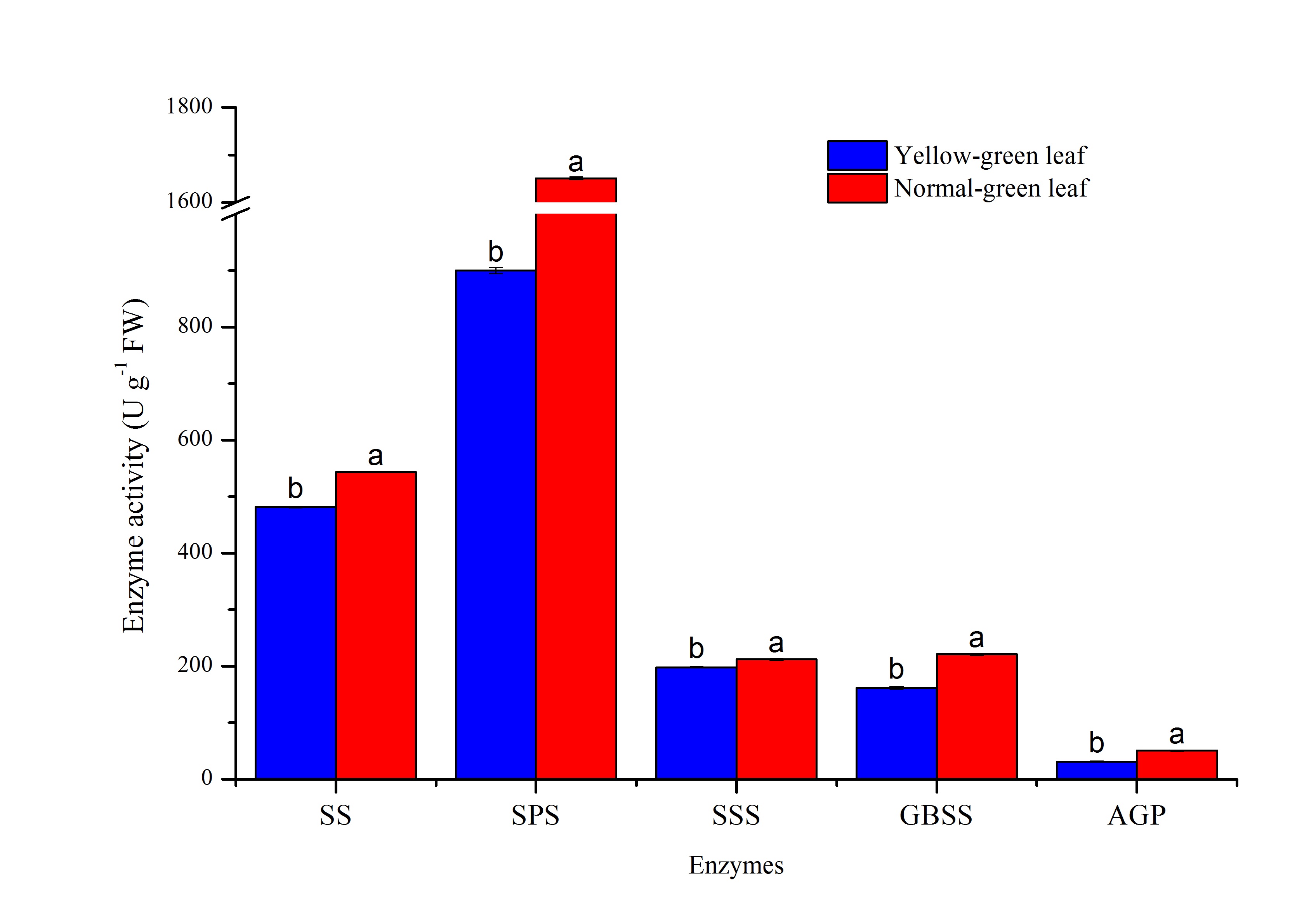

Supplement: Supplemental Information 8 — Lowercase letters a and b above the columns indicate differences between the yellow-green leaf mutant and the normal green leaf inbred line at P<0.05, according to least significant difference (LSD) tests. [file peerj-09-10567-s008.jpg]

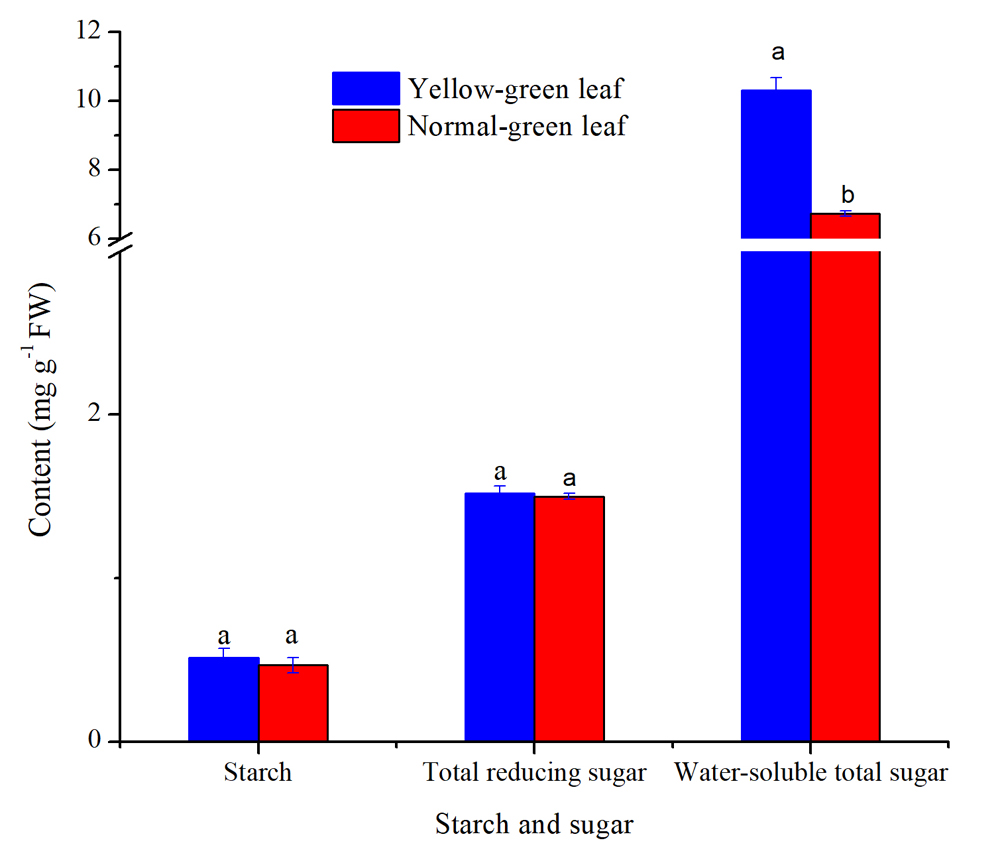

Supplement: Supplemental Information 9 [file peerj-09-10567-s009.jpg]

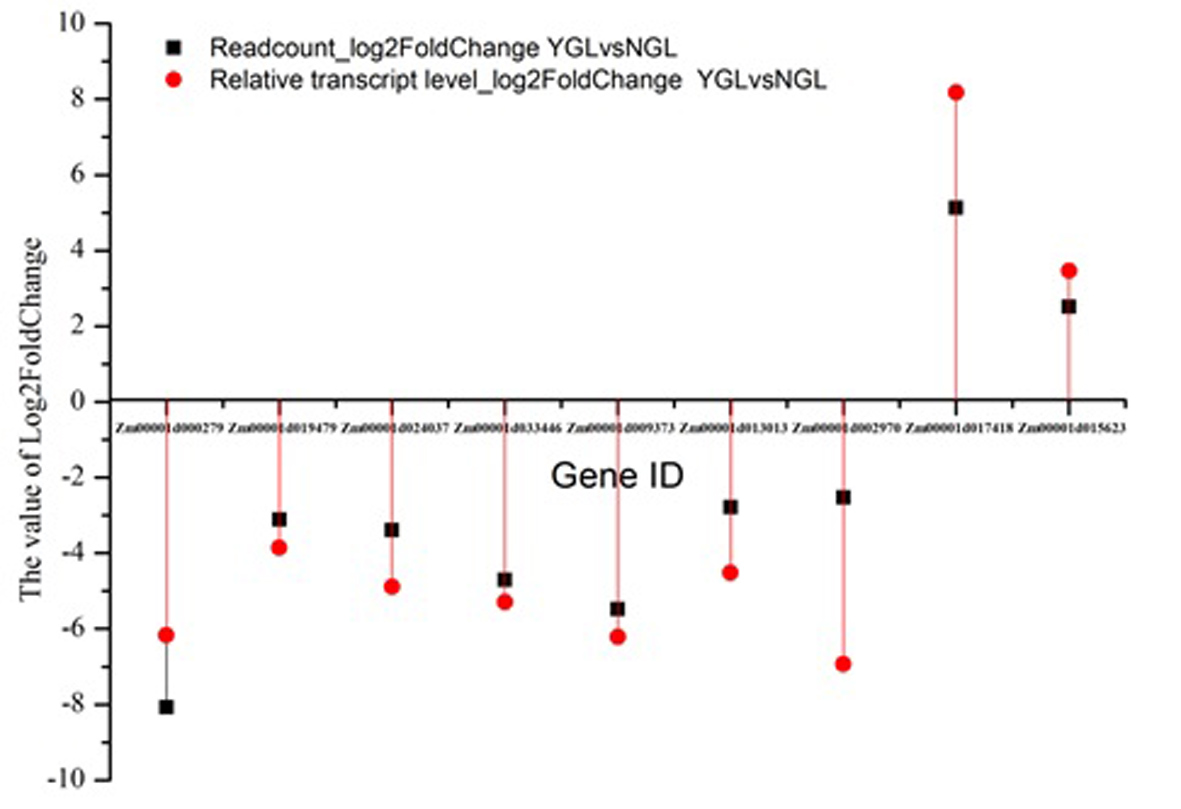

Supplement: Supplemental Information 10 [file peerj-09-10567-s010.jpg]
